# The emerging roles of E3 ubiquitin ligases in ovarian cancer chemoresistance

**DOI:** 10.20517/cdr.2020.115

**Published:** 2021-06-19

**Authors:** Yang Meng, Lei Qiu, Su Zhang, Junhong Han

**Affiliations:** Research Laboratory of Cancer Epigenetics and Genomics, Department of General Surgery, Frontiers Science Center for Disease-related Molecular Network, Cancer Center and National Clinical Research Center for Geriatrics, West China Hospital, Sichuan University, Chengdu 610041, China.; ^#^Yang Meng and Lei Qiu equally contributed to this manuscript.

**Keywords:** Ovarian cancer, chemoresistance, E3 ubiquitin ligases, inhibitor

## Abstract

Epithelial cancer of the ovary exhibits the highest mortality rate of all gynecological malignancies in women today, since the disease is often diagnosed in advanced stages. While the treatment of cancer with specific chemical agents or drugs is the favored treatment regimen, chemotherapy resistance greatly impedes successful ovarian cancer chemotherapy. Thus, chemoresistance becomes one of the most critical clinical issues confronted when treating patients with ovarian cancer. Convincing evidence hints that dysregulation of E3 ubiquitin ligases is a key factor in the development and maintenance of ovarian cancer chemoresistance. This review outlines recent advancement in our understanding of the emerging roles of E3 ubiquitin ligases in ovarian cancer chemoresistance. We also highlight currently available inhibitors targeting E3 ligase activities and discuss their potential for clinical applications in treating chemoresistant ovarian cancer patients.

## Introduction

### Ubiquitination in cancer

**Figure 1:**
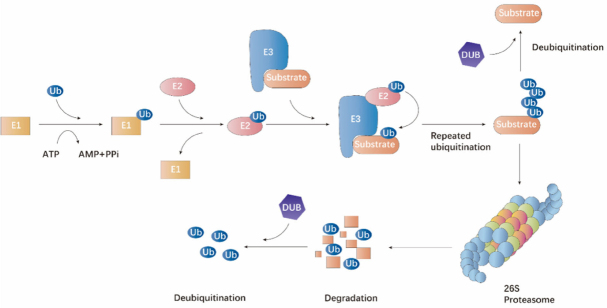
The ubiquitin-proteasome proteolytic pathway. The process of protein degradation via the ubiquitin-proteasome pathway is mediated by an organized milieu of activating (E1), conjugating (E2) and ligating (E3) enzymes to regulate the ligation of ubiquitin to the protein substrate. The substrate attached with multiple ubiquitin molecules are targeted to the 26S proteasome complex for degradation. Ubiquitin molecules can be removed by the action of deubiquitinating enzymes.

Ubiquitin is evolutionarily conserved and modifies proteins post-translationally for degradation or non-degradative signaling. It is evolutionarily conserved and is covalently coupled to lysine residues sequentially by activating (E1), conjugating (E2) and ligating (E3) enzymes^[1,2]^. The C-terminus of ubiquitin is activated by an E1 activating enzyme first and then transferred to the active site of an E2 conjugating enzyme [Figure 1]. Subsequently, an E3 ubiquitin ligase bridges the target protein and the E2-ubiquitin intermediate to catalyze isopeptide bond formation between the substrate lysine and the ubiquitin C-terminal glycine [Figure 1]^[3]^. The cellular functions of ubiquitination involve several complicated cellular processes, including proteasomal degradation of proteins, endocytosis, protein-protein interactions, intracellular trafficking, cell cycle progression, DNA repair, autophagy, inflammatory signaling, and modulation of enzymatic activity^[4]^. The deubiquitination process is also a crucial step mediated by ubiquitin recycling enzymes in proteasomal pathway, which is important for maintaining cellular ubiquitin homeostasis. Deubiquitinating enzymes (DUBs) cleave ubiquitin residues from protein substrates conjugated with ubiquitin and the covalent links between ubiquitin-ubiquitin [Figure 1]^[5]^. Similar with ubiquitin ligases, DUBs are also implicated in the adjustment of various cellular activities, including DNA repair, cell cycle progression, chromosome segregation, gene expression, and spermatogenesis^[5-7]^. Therefore, dysregulation of ubiquitination may lead to human organism disorders such as cancer.

The approval and effective clinical application of proteasomal inhibitors for the treatment of multiple myeloma^[8,9]^ has inspired researchers to use various aspects of the ubiquitination system as targets for treatment of malignant cancers as well as other diseases^[1]^. Advancement in technologies such as proteomic mass spectrometry and development of ubiquitin/ubiquitination-specific antibodies, have provided possibilities to trace ubiquitination precisely in a genome-wide manner^[1]^. Consequently, numerous studies have uncovered correlations between dysregulation of the ubiquitination-proteasome system and cancer. For example, some Cbl family ubiquitin ligases promote cancer by mediating lysosomal sorting and degradation of RTKs^[10]^. The E3 ligase c-Cbl is frequently dysregulated in myelodysplastic-myeloproliferative neoplasms and is also couped with myelodysplastic syndromes, myeloid neoplasms, and primary colorectal cancer^[1]^. Therefore, ubiquitination plays an important role in cancer.

### Ovarian cancer chemoresistance

GLOBOCAN estimated in 2018 that there would be nearly 300,000 newly diagnosed cases of ovarian cancer per year and around 185,000 would die from this cancer^[11]^. Ovarian cancer is the most deadly gynecological cancer. Because of lacking noticeable early symptoms, approximately 70% of the ovarian cancer patients are diagnosed in advanced stages and about 85% of the patients have cancer that already metastasized to other organs^[12,13]^. Apart from development of novel targeted therapies, such as PARP (poly ADP-ribose polymerase) inhibitors, and application of intraperitoneal delivery, the five-year survival rate of ovarian cancer patients with advanced cancer remains only 20%-40%^[14]^. At present, the standard therapeutic regimen for ovarian cancer patients is tumor debulking surgery followed by chemotherapy treatment with platinum derivative (cisplatin or carboplatin) combined with paclitaxel. This therapy manifests encouraging effects in many terminal stage epithelial ovarian cancer patients. However, the development of chemoresistance is a chief barrier against the improvement of ovarian cancer patient outcome^[15]^. Therefore, understanding the mechanisms of chemoresistance development is crucial for overcoming ovarian cancer treatment failure.

The mechanisms of ovarian cancer chemoresistance are remarkably elusive. Studies have uncovered that chemoresistance developed through several traditional mechanisms, including diminished drug uptake, increased drug efflux from cells^[16,17]^, modified drug target, upregulation of DNA repair systems, and enhanced replicative bypass of platinum-DNA adducts^[18]^, as well as diminished ratio of drug-induced DNA damage in the resistant cells^[19]^. In recent years, E3 ubiquitin ligases have been manifested to play key roles in chemoresistance through degradation of various chemoresistance-related substrates in ovarian cancer.

In this manuscript, we review the emerging roles of E3 ubiquitin ligases in ovarian cancer chemoresistance. We also summarize inhibitors that target E3 ubiquitin ligases which may help overcome chemoresistance and improve patient outcome. Finally, we highlight challenges and restrictions in targeting E3 ubiquitin ligases as ovarian cancer chemotherapy.

## Classification of E3 ubiquitin ligases

Human E3 ubiquitin ligases comprise more than 600 members and are identified in mammalian ubiquitination cascades^[20-22]^. E3 ubiquitin ligases are classified into three families based on differences in structure and function, mainly comprising the HECT (homologous to the E6-associated protein carboxyl terminus) domain family, the *RING* (really interesting new gene) finger family, and the RING in-between-RING (RBR) E3 ubiquitin ligases^[23,24]^.

There are roughly 30 HECT domain-containing E3 ligases in mammals. Among their many functions, HECT domain E3s determine ubiquitination specificity and play prominent roles in the trafficking of many receptors, regulating the immune response and several signaling pathways in cell proliferation^[25]^.

As the largest family of E3 ubiquitin ligases, substrate of RING-finger E3 ubiquitin ligases involve various cellular processes, including cell metabolism, cell proliferation, apoptosis, differentiation and DNA repair. RING-finger E3 ligases differ from the HECT domain E3s in that they do not form catalytic intermediates with ubiquitin. In contrast, the RING-finger domain serves as a platform that bridges the E2 conjugating enzyme and the substrate. Evidence shows that RING-finger domains can also activate E2 ubiquitin-conjugating enzymes allosterically^[26]^.

The RBR E3 ligases, a family of RING-HECT hybrids, contain an RBR module (RING1-IBR-RING2) comprising two RING domains (RING1 and RING2) connected via an in-between-RING (IBR) domain^[27,28]^. RBR E3 ubiquitin ligases differ from their RING-type E3 cousins mainly because they possess an active site, a feature not observed in other RING-type E3s^[29]^. Similar to canonical RING-type E3s, the RING1 domain of the RBR module binds ubiquitin-loaded E2 enzymes (E2-Ubs), whereas an essential active-site Cys in RING2 recruits ubiquitin from an E2-Ub to generate a covalent E3-Ub intermediate, which is otherwise only seen in HECT-type E3s^[29]^. RBR E3s are therefore regarded as RING-HECT hybrids. HHARI and Parkin were the first two RBR members revealed to have the hybrid mechanism^[29]^. TRIAD1, RNF144A, HOIP, and HOIL-1L have later been verified with this active-site Cys for catalytic activity^[30-33]^.

E3 ubiquitin ligases play crucial roles in the ubiquitin-proteasome pathway, thus may be critical to cancer progression and chemoresistance. Indeed, multiple studies have indicated prominent roles of E3 ubiquitin ligases in ovarian cancer chemoresistance.

## E3 ubiquitin ligases in ovarian cancer chemoresistance

### The HECT domain E3 ligases in ovarian cancer chemoresistance

**Figure 2:**
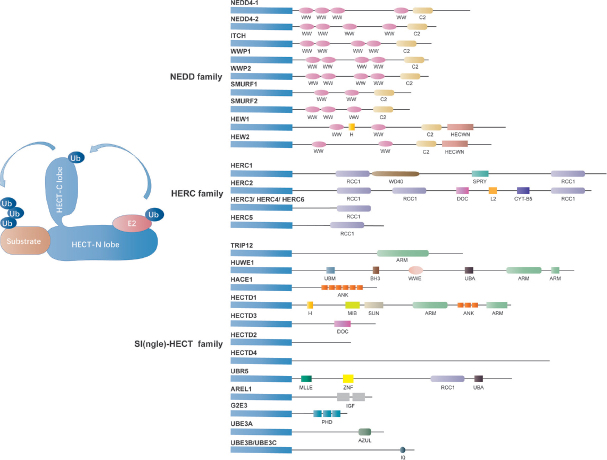
Classification of the HECT domain E3 ligases. The mammalian HECT E3 ligases have been classified into three subgroups, including HERC family (HERC E3s containing RLDs family, 6 members), NEDD4 family (C2-WW-HECT E3s possessing WW domains family, 9 members) and SI(ngle)-HECT family [SI(ngle)-HECT E3s lacking either WW or RLDs domains family, 13 members] [Figure 1]. The HECT domain is comprised of a smaller C-terminal lobe consisting of the active-site cysteine residue and a larger N-terminal lobe including the E2-binding site. The conjugation between ubiquitin with a protein substrate consists of three steps: activated ubiquitin binding to a specific cysteine residue of one of several E2s, loading ubiquitin on themselves through the formation of a ubiquitin-thioester intermediate with the catalytic cysteine located at the C terminus of the HECT domain, and immediately deliver of ubiquitin to the protein substrates.

The HECT family consists only 28 of the 600 members of human E3 ligases^[34]^. Based on the protein-protein interaction domains that determine their substrate specificity, the HECT-type E3s can be further classified into three subfamilies: C2-WW-HECT E3s possessing WW (tryptophan-tryptophan) domains, HERC E3s containing RLDs (RCC1-like domains), and SI(ngle)-HECT E3s lacking either WW or RLDs domains [Figure 2]^[25,35]^. Currently, the most well studied HECT domain E3 ligases that relate to chemoresistance are ubiquitin protein ligase E3 component n-recognin 5 (UBR5/EDD ), HECT, UBA, and WWE domain containing 1 (HUWE1/MULE) and HECT-Type E3 Ubiquitin Transferase Itchy Homolog (ITCH ), among which UBR5/EDD and ITCH have been reported to be implicated in ovarian cancer chemoresistance.

#### NEDD4 family

The NEDD4 family of well-characterized HECT domain-containing E3 ubiquitin ligase are highly evolutionarily conserved from yeast to humans^[36]^. As shown in Figure 2, the NEDD4 family contains nine mammalian members in humans, including NEDD4 (NEDD4-1), NEDD4-2 (NEDD4L), WW domain-containing E3 ubiquitin protein ligase 1, WWP2/atropine-1-interacting protein 2 (AIP2), SMAD-specific E3 ubiquitin protein ligase 1 (SMURF1), SMURF2, ITCH/atropine-1 interaction protein 4 (AIP4), NEDL1 (HECW1), and NEDL2 (HECW2)^[37]^. The structure of NEDD4 family E3 ligase consists of a catalytic C-terminal HECT domain for Ub protein ligation, 2-4 WW domains and an N-terminal C2 domain as a Ca^2+^ or phospholipid-binding motif for the cell membrane substrate binding^[38,39]^. Recent studies have identified that NEDD4 family enzymes have been demonstrated to play a key role in the progression of various cancers and drug resistance in cancer therapy. One study showed significantly reduced NEDD4-2 protein expression in invasive ovarian epithelial cancer tissues compared to ovarian non-cancer tissue^[40]^. In addition, NEDD4 is highly expressed in erlotinib-resistant NSCLC cells and cisplatin-resistant nasopharyngeal carcinoma cells^[41]^. Nonetheless, upregulation of NEDD4 resensitized multiple myeloma cells to bortezomib treatment via ubiquitination of Akt and degradation of pAkt-Ser473, while downregulation of NEDD4 resulted in bortezomib resistance in multiple myeloma cells^[42]^. What’s more, Akkaya *et al*.^[43]^ also demonstrated NEDD4 as a ubiquitin ligase for ATP binding cassette subfamily B member 1, which is a multidrug resistance pump that mediates drug resistance, indicating that NEDD4 directly participates in drug resistance. These pieces of evidence suggest an important role of NEDD4 in the regulation of chemoresistant ovarian cancer.

#### ITCH

ITCH is classified as a NEDD4 family E3 ligase with a C-terminal HECT domain for E3 ligase activity and WW domains for substrate recruiting^[44]^. However, the intramolecular interaction of ITCH catalytic HECT domain with its WW domains blocks its ubiquitination activity^[45]^.ITCH regulates the transcriptional activity of several transcription factors including NF-kB1 and JUNB, probably playing an important role in inflammatory signaling pathways. ITCH forms a complex with FLIP (FLICE-like inhibitory protein) and mediates its degradation and tumor necrosis factor α-induced apoptosis^[46,47]^. Cisplatin treatment induces ITCH-FLIP-p53 interaction, colocalization and FLIP degradation in chemosensitive but not chemoresistant ovarian cancer cells^[48]^. p53-FLIP interaction and FLIP ubiquitination can also be facilitated by inhibiting Akt function^[48]^. Abedini *et al.*^[48]^ further demonstrated that gelsolin was highly expressed in chemoresistant ovarian cancer cells and cisplatin failed to abolish the intact gelsolin-FLIP-ITCH interaction, leading to the dysregulation of the downstream responses.

#### UBR5/EDD

The ubiquitin protein ligase E3 component N-recognin 5 (UBR5, also known as EDD) belongs to the SI(ngle)-HECT family of E3 ubiquitin ligases that are involved in DNA damage response by suppressing RNF168, an E3 ligase that mediates the accumulation of ubiquitynated-H2A and -H2AX at DNA damage sites, and G2 checkpoint control^[49-52]^. UBR5 amplification in mRNA levels has been demonstrated in ovarian and breast cancer^[53]^. UBR5 is considered as an undesirable prognostic factor for patients with serous epithelial ovarian cancer due to its modulation in cisplatin resistance^[54]^. Bradley *et al.*^[55]^ also reported that UBR5 enhanced cell survival and cisplatin resistance and might serve as a therapeutic target for epithelial ovarian cancer. They further showed that UBR5 might have modulated ovarian cancer cell survival through regulating expression of the prosurvival protein myeloid cell leukemia sequence 1 (Mcl-1)^[55]^. As a novel ubiquitin ligase for the proapoptotic protein modulator of apoptosis protein 1 (MOAP-1, also known as MAP-1), UBR5 influences ovarian cancer cell cisplatin resistance by mediating MOAP-1 ubiquitination and degradation through cooperation with Dyrk2 kinase^[56,57]^. UBR5 upregulation in recurrent and platinum-resistant ovarian cancers indicates that targeting UBR5 may be an effective strategy for chemoresistant ovarian cancer treatment^[57]^.

### RING-finger domain E3 ligases in ovarian cancer chemoresistance

RING-finger domain E3 ubiquitin ligases contain either a domainor subunit with a RING motif. They can be either monomeric or members of multi-subunit E3 ligase complexes^[58]^. Previous evidence indicates that the RING-domain E3 ubiquitin ligases including Cullin-RING E3 ligase complexes such as SCF complex (SKP1, Cullin and F-box protein), the APC/C complex, as well as monomeric XIAP, MDM2, MUL-1, Pirh2, and SIAH2 may all be associated with ovarian cancer chemoresistance.

#### Cullin-RING E3 ubiquitin ligases

**Figure 3:**
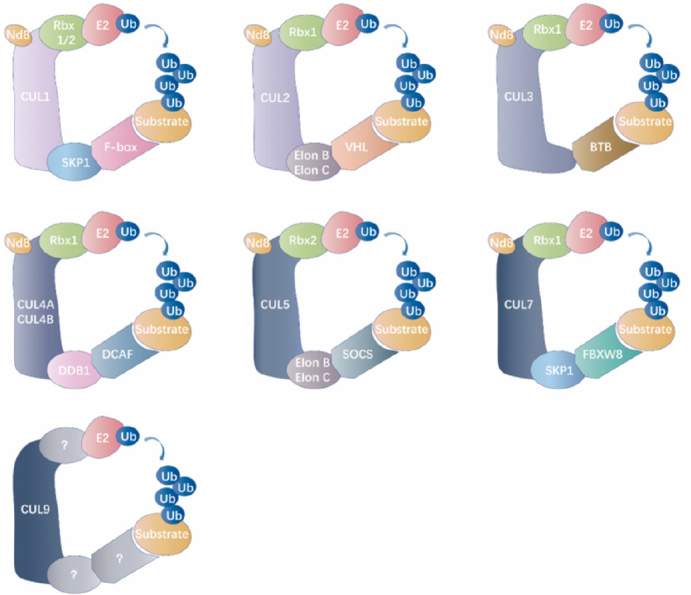
Classification of the cullin-RING E3 ubiquitin ligases. The general catalytic core of cullin-RING ligases (CRLs) is comprised of a RING protein and a cullin-family member, which delimits this modular category of RING ubiquitin ligase. NEDD8 (Nd8, neural precursor cell expressed developmentally down-regulated protein 8), as a ubiquitin-like modifier, efficiently activates Cullins by the covalent conjugation. Each Cullin exploits modular assembly to enroll different substrates to a general catalytic core by altering its substrate receptors. Cullin family members from different organisms recruit RING-finger proteins (Rbx1/2), adaptor proteins (SKP1 for CUL1/7, Elon B/Elon C for CUL2/5, BTB for CUL3, and DDB1 for CUL4A/B) and receptor proteins (F-box proteins for CUL1, VHL for CUL2, DCAF proteins for CUL4A/B, SOCS protein for CUL5, and FBXW8 protein for CUL7) for assembling multi-subunit complexes into ubiquitinated specific substrate proteins. However, the assembly of the CRL9 complex is ambiguous. Nd8: NEDD8; Ub: ubiquitin; CUL: cullin; SKP1: S-phase kinase-associated protein 1; VHL: von Hippel-Linda; BTB: broad complex, tramtrack, ‘bric-a-brac’; CRL: cullin RING ligase; DCAF: DDB1 CUL4 associated factor; DDB1: DNA damage-binding protein 1; RING: really interesting new gene; SOCS: suppressors of cytokine signalling.

Cullin-RING E3 ubiquitin ligases are the most extensively studied subfamily of multi-subunit complex RING ligases [Figure 3]. Cullin-RING ligase complexes are generally comprised of four indispensable subunits that include a Cullin scaffold (Cullin 1, Cullin 2, Cullin 3, Cullin 4A, Cullin 4B, Cullin 5, Cullin 7, or Cullin 9), a RING-finger protein (RBX1 or RBX2), an adaptor protein (SKP1, Elongin B, Elongin C, or DDB1), and a substrate recognition protein (over 400 known substrates)^[59-62]^. As the central coordinator in forming the Cullin-RING E3 complex, the Cullin scaffold provides a platform bridging the RING finger subunit with the adaptor subunit^[61]^. The RING-finger subunit acts as a docking site for the substrate recognition module and E2 to promote transfer of ubiquitin from E2 to the recruited substrate. The substrate recognition module interacts with the adaptor protein and determines substrate specificity^[61,63]^. Cullin-RING E3 ubiquitin ligases are responsible for approximately 20% of cellular ubiquitination events conducted by ubiquitin-proteasome system^[64,65]^.

The SCF E3 ubiquitin ligase complex is a member of the Cullin-RING family and consists of CUL1, SKP1, and F-box proteins^[63]^. One of the typical F-box proteins β-TRCP promotes protein degradation by recognizing the phosphorylation of many key signaling molecules and plays a crucial role in cell cycle regulation, DNA damage response, and cancer development^[66,67]^. Extensive studies have demonstrated that AEBP2 (a zinc finger protein) ubiquitination and destruction mediated by SCF-β-TRCP ubiquitin ligase complex control cisplatin resistance in ovarian cancer^[68]^.

FOXO5 overexpression has been reported in ovarian cancer^[69]^. As a tumor suppressor and a *TGF-β*/*SMAD4* target gene, methylation of FBXO32 promoter correlates with poor ovarian cancer prognosis while reintroduction of FBXO32 can sensitize the cells to cisplatin treatment^[70]^. FBXL7 plays a prominent role in ovarian cancer survival and its overexpression in ovarian cancer patients is implicated in paclitaxel resistance and poor prognosis^[71,72]^. Although the exact mechanism regarding how FBXL7 influences ovarian cancer chemoresistance has not yet been well clarified, a study in gastric cancer suggests that FBXL7 adjusts survivin expression through the ubiquitin-proteasome pathway^[73]^. AURKA (auroral kinase A ) down-regulates FBXL7 and subsequently inhibits survivin degradation and increases survivin protein level, leading to enhanced drug resistance^[73]^. FBXL10, also known as Ndy1, JHDM1B or KDM2B, was initially characterized as a H3K36 demethylase, which controls cell proliferation and senescence by regulating p15^[74,75]^. Yan *et al*.^[76]^ demonstrated that mirR146b sensitized ovarian cancer cells to paclitaxel and cisplatin and enhanced cell proliferation by inhibiting FBXL10.

Jazaeri *et al*.^[13]^ manifested that CUL3 interacted with KEAP1 (kelch-like erythroid cell associated protein-1) and RBX1 (RING-box 1) to form the CRL3-KEAP1 ubiquitin ligase, while inhibition of CRL3 sensitized both Skov3 and ES2 cells to cisplatin.

CRL4^CDT2^ repression and CDT1 accumulation are critical biochemical events contributing to the genotoxic effects of MLN4924 in ovarian cancer cells [Table 1]^[62]^. Moreover, VPRBP is upregulated in high-grade ovarian patient tumors and CRL4^VPRBP-DCAF1^ regulates the degradation and nonproteolytic activation of the cell transcription factor Foxm1^[77]^. Our previous study has also illustrated that CRL4^Cul4/DDB1^ E3 ubiquitin ligase regulates ovarian cancer drug resistance by targeting the anti-apoptotic protein BIRC3^[78]^. Jang *et al*.^[79]^ further showed that deficiencies in RepID, CRL4, or RBBP7 delayed mitotic exit, increased genomic instability, and enhanced sensitivity of ovarian cacner cells to paclitaxel.

**Table 1:** E3 ligase and their impact on cancer drug resistance

Classification	E3 ligase			Targeted ubstrate	Associated pathway	Drug resistance	Inhibitor	Ref.
HECT E3 ligases	UBR5/EDD			MOAP-1	Intrinsic apoptosis pathway	Cisplatin		[54,57]
	ITCH			FLIP		Cisplatin		[46,153]
	NEDD4			ABCB1				[43]
RING-Domain E3 ligases	Cullin-RING E3 ligases	SCF ubiquitin ligase	β-TRCP	AEBP2	Cell proliferation	Cisplatin		[68]
			FBxo32		Cell proliferation	Cisplatin		[70]
			FBXL7	Survivin*	Ubiquitin proteasome pathway	Cisplatin/paclitaxel		[71-73]
			FBXL10	P15*	Cell proliferation	Cisplatin/paclitaxel		[74,75]
		CRL3	KEAP1	Nrf2*	Cell survival	Cisplatin	MLN4924	[13]
		CRL4	CDT2/DCAF2	CDT1	Cell survival and proliferation	Cisplatin	MLN4924	[62]
			VPRBP-DCAF1	Foxm1*	Cell cycle			[77]
					STAT1-STAT3 pathway	Cisplatin	MLN4924	[78]
RING-domain E3 ligase	XIAP			P53	Caspase3 PI3K-AKT pathway	Cisplatin		[85,88]
	MDM2			P53	Apoptosis pathway	Cisplatin	Nutlins	[97]
	MUL-1			AKT	PI3K-AKT pathway	Cisplatin		[111]
	APC/C			PLK1	Apoptosis pathway, mitotic	Cisplatin/paclitaxel		[81-83]
	Pirh2			Twist1	EMT	Cisplatin		[113,114]
	Siah2			HIF-1α*	Hypoxia, MAPK signaling	Platinum	Menadione	[119,126,127]
RBR ligase	RNF31/HOIP			Caspase3/Caspase8*	JNK pathway	Cisplatin		[24,131]

The substrate proteins with asterisk mark may be involved in the regulation of ovarian cancer cisplatin resistance.

Given the emerging role of Cullin-RING ubiquitin ligases in ovarian cancer chemoresistance, targeting the activity of Cullin-RING complexes may be an efficacious anticancer strategy for targeted ovarian cancer therapy.

#### Multi-subunit RING E3 ligase - APC/C

The APC/C (anaphase-promoting complex/cyclosome) is the major ubiquitin ligase involved in the regulation of mitosis^[80,81]^. Raab *et al.*^[82]^ reported that by eliminating the anti-apoptotic shielding through MCL-1 inhibition and blocking the activity of APC/C, the apoptosis-resistant and slippage prone HGSOCs were sensitized to the frontline therapy including paclitaxel. Belur Nagaraj *et al*.^[83]^ uncovered that long-term cisplatin treatment induced mitotic exit vulnerability in the presence of APC/C dysfunction along with cisplatin-resistance, where APC/C^CDC20^ inhibition increased the sensitivity of pharmacologic PLK1 inhibition, which in turn diminished cisplatin-resistant cell survival and aggravated spindle checkpoint response in cisplatin resistant ovarian cancer cells.

#### XIAP

As a member of the inhibitor of apoptosis protein family, X-linked inhibitor of apoptosis protein (XIAP) contains three baculoviral inhibitory repeat domains at the N-terminus, which are associated with direct caspase inhibition^[84]^. XIAP also comprises a C-terminal RING-finger domain with E3 ubiquitin ligase activity through the NEDD8 conjugation pathway, targeting effector caspases for neddylation and inactivation^[85]^. XIAP does not only regulate apoptosis, but also influence mitogenic kinase signaling, copper homeostasis, inflammatory signaling and immunity, as well as tumor metastasis. It has previously been reported that cisplatin down-regulates XIAP in chemosensitive ovarian cancer cells, whereas chemoresistant cells are not affected^[86]^. Furthermore, XIAP determines chemoresistance, since its downregulation in chemoresistant cells sensitizes cells to the cytotoxicity induced by cisplatin, whereas its overexpression in chemosensitive cells can lead to chemotherapy tolerant phenotypes^[86,87]^. Inhibition of p53-mediated cell death and upregulation of the PI3K/Akt pathway partially contribute to the anti-apoptotic effects of XIAP^[88]^.

XIAP itself can be ubiquitinated and degraded through the 26S proteasome under specific cellular condition. One study suggests that XIAP is a potential substrate of HtrA1 and the HtrA1-mediated XIAP ubiquitination and degradation is sufficient to sensitize cells to chemotherapy, proposing that restoring the expression of HtrA1 would be a prospective treatment strategy against ovarian cancer chemoresistance^[89]^. Cisplatin induces XIAP content decrease and cytosolic HtrA2/Omi level increase in cisplatin-sensitive ovarian cancer cells^[90]^. In addition, HtrA2/Omi level change correlates with the XIAP downregulation, caspase-3 activation, and apoptotic response, suggesting that cisplatin resistance probably due to XIAP neutralizing caspase-3 activation and lower cytosolic HtrA2/Omi level in response to cisplatin in human ovarian cancer cells^[90]^.

#### MDM2

As a RING finger dependent E3 ubiquitin ligase, murine double minute 2 (MDM2, also known as HDM2) plays crucial roles in regulating p53 protein ubiquitination and degradation^[91-96]^. In addition, it is a component of the Trim28-ERBB4-MDM2 complex linking growth factor and DNA damage repair pathways. Recently, MDM2 inhibitors have been assessed in clinical trials. Mir *et al.*^[97]^ suggest that MDM2 antagonists induce apoptosis and are able to overcome chemoresistance in TP53 wild-type ovarian cancer cells when synergized with cisplatin. The authors used small molecules called nutlins to inhibit the binding of p53-MDM2 and induce apoptosis in chemoresistant ovarian cancer by activating p53 pathway^[97]^. Meanwhile, Wu *et al*.^[98]^ demonstrated that F14 overcame cisplatin resistance of HGSOC (high-grade serous ovarian cancer) by accelerating MDM2-regulated p53-R248Q ubiquitination and degradation . More recently, the research of Cui *et al.*^[99]^ indicated that overexpression of CCDC69 protein activated p14^ARF^-MDM2-p53 pathway and increased the cisplatin sensitivity in chemoresistant ovarian cancer. Intriguingly, the expression of CCDC69 protein extended the half-life of p53 and p14^ARF^ proteins and shortened the half-life of MDM2 protein^[99]^.

#### MUL-1

MUL-1 (mitochondrial E3 ubiquitin protein ligase 1, also known as MULAN^[34]^, MAPL^[100,101]^, GIDE^[102]^, and HADES^[103,104]^) exhibits weak E3 ubiquitin-protein ligase activity^[105,106]^, and it is one of the three known mitochondrial E3 ubiquitin ligases involved in mitophagy^[107,108]^, apoptosis^[101,102,108]^, and innate immune response^[109,110]^. Studies uncovered that metformin conducted its antitumor activity by downregulating AKT protein expression via MUL-1 E3 ligase^[111]^. These findings suggest that MUL-1 regulates metformin-mediated AKT degradation and the potential of using metformin as a therapeutic strategy in treatment against chemoresistant ovarian cancer cell.

#### Pirh2

Pirh2 (p53-induced RING-H2 protein) was initially identified as an ARNIP (androgen receptor N-terminal-interacting protein), which displayed ubiquitin ligase activity toward p53, p73, HDAC1, and CDKN1B^[112]^. Pirh2, along with Mdm2, was later found to be one of the major ubiquitin ligases targeting p53, preferentially acts on tetrameric p53, leading to proteasomal degradation^[113,114]^. Yang-Hartwich *et al*.^[115]^ showed that p53-Pirh2 complex promoted Twist1 degradation and inhibited EMT (epithelial-mesenchymal transition) in ovarian cancer. Since EMT is a critical process involved in cancer metastasis and chemoresistance, targeting Pirh2 may be a promising strategy in chemoresistance therapy for ovarian cancer.

#### SIAH2

Seven in absentia homolog 2 (SIAH2) is an evolutionarily conserved RING-finger ubiquitin ligase. SIAH2 has been defined as a central regulator of tumorigenesis and tumor progression by mediating ubiquitination of diverse cellular substrates. SIAH2 has a catalytic RING domain on its N-terminus followed by two zinc fingers and a C-terminal substrate-binding domain^[116]^. Increasing evidence reveals that SIAH2 proteins are also involved in multiple cellular processes, including transcription regulation, cell cycle, survival, hypoxic response, cellular clock function, cynaptic vesicle function in neurons, and mitochondrial biogenesis^[117-119]^. SIAH2 protein contributes to the progression of various malignant tumors, including breast carcinoma^[120]^, lung^[121]^ and prostate tumors^[122]^, and oral cancer^[123]^.

Some recent findings may explain the mechanisms through which SIAH2 mediates ovarian cancer chemoresistance. Qiu *et al.*^[124]^ demonstrated that SIAH2 expression was an independent factor for platinum chemotherapy resistance in patients with epithelial ovarian cancer, suggesting that SIAH2 protein plays an key role in chemotherapy resistance. Another study manifested that the levels of HIPK2 and DYRK2 were significantly elevated due to inhibition of SIAH2, leading to increased sensitivity of cells to DNA damage-induced apoptosis^[125]^. Shah *et al*.^[126]^ indicated that SIAH2 inhibitor menadione could be used as an efficacious therapeutic strategy for melanoma via weakening hypoxia and MAPK signaling [Table 1]. Furthermore, SIAH2 can sufficiently improve responses to chemotherapy by attenuating HIF-1α-mediated angiogenesis and hypoxia signaling^[127]^. SIAH2 may be reversely regulated by E2 and EGF, thereby causing drug resistance in cancer^[127]^. These data indicate that SIAH2 may be a potential biomarker in evaluating tumor chemoresistance and poor clinical outcomes in patients with epithelial ovarian cancer.

### The RBR E3 ligases-RNF31 in ovarian cancer chemoresistance

RING finger protein 31 (RNF31, also named HOIP) is the catalytic subunit of the E3 ubiquitin ligase LUBAC (linear ubiquitin assembly complex), which also consists of the components SHARPIN and HOIL-1L and regulates cisplatin-induced genotoxicity^[128,129]^. There is compelling evidence from large-scale gene-drug association studies that *RNF31* overexpression may contribute to cisplatin resistance in cancer^[130]^. Mackay identified RNF31 as a key regulator in response to cisplatin induced genotoxicity. Due to the potentiated apoptotic response, RNF31 deletion may enhance the cytogenetic toxicity of cisplatin in some cancer cell lines^[131]^. Cells deficient in RNF31 E3 ligase complex were cisplatin-sensitive due to a significant raise in caspase-3/8-mediated apoptosis and enhanced cisplatin-induced JNK activity. Moreover, evidence also suggests that cisplatin resistant ovarian cancer cells could recover sensitivity with shRNF31 knockdown^[24]^. These results imply that RNF31 is a potential target for developing platinum-based anticancer drugs in combinational chemotherapy.

## Conclusion and perspective

Research on E3 ubiquitin ligases has been gradually carried out in the field of chemotherapy resistance. As described above, some E3 ubiquitin ligases are promising targets for cancer therapies. Due to the specificity requirement of targeted therapy, inhibitors specifically targeting E3s may be highly specific drugs with few side effects. New methods with small molecule inhibitors against E3 ligase are considered to be suitable treatment strategies.

Proteolysis-targeting chimera (PROTAC), a useful technology for targeted protein degradation in cancer therapy, has been applied in the degradation of critical oncoproteins involved in malignant neoplastic disease, especially in hematological malignancies^[132]^. A PROTAC molecule is generally composed of a covalently linked ligand of an E3 ubiquitin ligase and a ligand (mostly small-molecule inhibitor) of the protein of interest. PROTAC binds to the protein of interest to recruit E3 ubiquitin ligase which in turn proximity-induce ubiquitination of the protein of interest and then degradation by endogenous 26S proteasomes^[132]^. The BRD4 of bromodomain and extra-terminal domain (BET) proteins had been indicated to play a significant role in the progression of diverse cancers, such as ovarian cancer, acute myeloid leukemia, prostate cancer and Burkitt’s lymphoma^[133]^. A recent study reveals that the anti-tumor activity of BET-PROTACs contributes to diminish cancer growth in an *in vivo* mouse xenograft model with cells resistant to bromodomain and extraterminal domain inhibitors, and are also very active in triple-negative breast cancer and ovarian cancer cell lines^[134]^. This suggests that PROTAC technology has a prominent potential in the perspective of ovarian cancer therapy.

The ubiquitination activity of Cullin-RING E3 ligases requires the ubiquitin-like protein NEDD8, which covalently binds the E3 ligase cullin component, to perform normal functions. Compound MLN4924 is a small molecule inhibitor specifically targeting NEDD8 activation [Table 1]. MLN4924 has been shown to effectively block tumor cell proliferation in multiple preclinical models^[135]^. Phase I clinical trial of MLN4924 for non-hematologic malignancies has been completed, whereas other trials using MLN4924 in a range of solid tumors and hematologic malignancies are under way or planned (NCT00677170, NCT00911066). Bortezomib, the proteasome inhibitor, suggests the prospect of using specific E3 inhibitors in anticancer therapy. Studies demonstrated that inhibiting Hdm2 along with bortezomib sensitized cells to bortezomib and overturned bortezomib resistance^[136]^. Many compounds, including nutlin-3 (RO5045337), serdemetan, and NSC-207895, have all demonstrated *in vitro* anticancer activities^[137-139]^. Nutlin-3 may be used as a promising targeted therapy agent and has been registered for Phase I trials for an series of malignancies (NCT00559533, NCT00623870), although results from these studies have not yet been published. Recent studies have shown that nutlin-3 plays a key role in drug resistance by targeting the interaction between p53 and HDM2 [Table 1]. It induces apoptosis in chemoresistant ovarian cancer by reactivating p53 pathway as a small molecule regulator for p53^[96]^. Furthermore, Shah *et al*.^[126]^ also suggest that a small inhibitor of SIAH2, menadione, can be used as an effective therapeutic strategy for melanoma by weakening hypoxia and MAPK signaling [Table 1]. PARK2, a RING-between RING-type E3 ubiquitin ligase, has been implicated in various cellular activities including mitochondria homeostasis, stress response, protein turnover, xenophagy^[140]^, and metabolism. Frequent PARK2 inactivation has been recognized in diverse cancers. The copy number loss and loss of heterozygosity of Park2 are found in ovarian cancer^[141]^, pancreatic adenocarcinoma^[142]^, and breast cancer^[141]^. Increasing evidence suggest that both Park2 and SPOP are associated with regulating PD-L1 stability, illustrating the need to excavate the roles of E3 ligases in animal models and clinic trial^[143]^. Given the recent success of immuno-oncology and CAR-T cell therapy, a further understanding of how E3 ligases affect macro-level phenotypes, such as tumor sensitivity to immunotherapies, may influence the design of clinical therapies.

The ubiquitination process is a dynamic process that is orchestrated by E3 ubiquitin ligases and DUBs. Increasing evidence support that DUBs are differentially expressed in numerous cancers such as pancreatic ductal adenocarcinoma, ovarian cancer, non-small cell lung cancer, and breast cancer^[144,145]^. Based on recent data, alterations of DUB activities are also involved in various pathological disorders consisting of malignant neoplastic diseases and multiple neurological disorders. Several DUBs have been identified as regulators in the development of ovarian cancer, including USP1, USP7, USP5, USP39, and UCHL1^[146]^. One study shows that ubiquitin carboxyl terminal hydrolase 1 is a putative tumor suppressor in ovarian cancer cell lines and contributes to ovarian cancer cisplatin resistance^[147]^. Intriguingly, Sonego *et al*.^[148]^ indicated that Snail was a USP1 target, opening the way to a novel strategy in overcoming platinum resistance and more successfully treating patients with ovarian cancer.These data suggest that DUBs may be explored as a promising therapeutic target for treatment of ovarian cancer and other diseases.

Increasing amount of evidence suggests that non-coding RNAs (ncRNAs) play important roles in most cancers cellular network and functions. Dysregulation of ncRNAs in cancer has also been reported to renovate drug responsiveness in chemoresistant cancer cells. Recent data showed that interaction between long non-coding RNA GHET1 (gastric carcinoma high expressed transcript 1) and the E3 ubiquitin ligase VHL (von Hippel-Lindau) blocked VHL-mediated degradation of HIF1α (hypoxia-inducible factor-1α) and enhanced the protein level of HIF1α, which then promoted the proliferation of ovarian cancer cells^[149]^. Hu *et al.*^[150]^ found that E3 ubiquitin-protein ligase MARCH7 (membrane-associated RING finger protein 7) interaction with MALAT1 modulates ATG7 by competing with miR-200a and promoting autophagy, metastasis and invasion in ovarian cancer. Another study found miR-96 boosting apoptosis through reduction of the antiapoptotic regulator XIAP and the p53 stability regulator UBE2N (ubiquitin-conjugating enzyme E2N), regulating 5-FU sensitivity in colorectal cancer cells^[151]^. Furthermore, it is confirmed that miR-133b may reduce ovarian cancer chemotherapy resistance by silencing the expression of the drug-resistance-related proteins, glutathione S-transferase-π and multidrug resistance protein 1^[152]^. In the future, the combination of ncRNAs with chemotherapy agents may show exciting efficacy in overturning chemotherapy resistance in ovarian cancer.

Broadly speaking, E3 ligases play important roles in mediating drug resistance. With the development and advancing of proteomics technology, more and more substrate proteins have been identified. Theoretically, the most efficacious treatment for the ubiquitin proteasome pathway is to block the E3 ligase at specific substrate recognition sites. However, due to the extreme complexity of the candidate small molecule drugs, blocking protein-protein interaction is very difficult. At present, many treatment strategies are still in large-scale experimental studies. How to overcome clinical drug resistance through regulating the interaction between key signal transduction factors and E3 ligases is still the focus of current research and the most difficult issue. Further understanding of the mechanisms underlying E3-substrate interaction and function will provide a good theoretical base for clinically targeting E3 ligases for chemotherapy against drug resistant ovarian cancer in the future.

## Declarations

### Copyright

© The Author(s) 2021.
